# RING-finger E3 ligases regulatory network in PI3K/AKT-mediated glucose metabolism

**DOI:** 10.1038/s41420-022-01162-7

**Published:** 2022-08-24

**Authors:** Wenke Wang, Bei Shi, Ruiting Cong, Mingjun Hao, Yuanyuan Peng, Hongyue Yang, Jiahui Song, Di Feng, Naijin Zhang, Da Li

**Affiliations:** 1 Center of Reproductive Medicine, Shengjing Hospital of China Medical University, Shenyang, China; 2 Department of Physiology, School of Life Sciences, China Medical University, Shenyang, China; 3 Education Center for Clinical Skill Practice, China Medical University, Shenyang, China; 4 Department of Cardiology, the First Hospital of China Medical University, Shenyang, Liaoning China

**Keywords:** Ubiquitylation, Ubiquitylation

## Abstract

The phosphatidylinositol 3-kinase (PI3K)/AKT signaling pathway plays an essential role in glucose metabolism, promoting glycolysis and resisting gluconeogenesis. PI3K/AKT signaling can directly alter glucose metabolism by phosphorylating several metabolic enzymes or regulators of nutrient transport. It can indirectly promote sustained aerobic glycolysis by increasing glucose transporters and glycolytic enzymes, which are mediated by downstream transcription factors. E3 ubiquitin ligase RING-finger proteins are mediators of protein post-translational modifications and include the cullin-RING ligase complexes, the tumor necrosis factor receptor-associated family, the tripartite motif family and etc. Some members of the RING family play critical roles in regulating cell signaling and are involved in the development and progression of various metabolic diseases, such as cancer, diabetes, and dyslipidemia. And with the progression of modern research, as a negative or active regulator, the RING-finger adaptor has been found to play an indispensable role in PI3K/AKT signaling. However, no reviews have comprehensively clarified the role of RING-finger E3 ligases in PI3K/AKT-mediated glucose metabolism. Therefore, in this review, we focus on the regulation and function of RING ligases in PI3K/AKT-mediated glucose metabolism to establish new insights into the prevention and treatment of metabolic diseases.

## Facts

1. PI3K/AKT signaling can directly or indirectly alter glucose metabolism, which is a crucial component of mammalian organ function.
2. The mechanisms of RING-finger E3 ligases in PI3K/AKT-mediated glucose metabolism are widely researched.
3. Zinc-binding RING-finger adaptor proteins such as CRLs, TRAF, TRIM, and MARCH play a significant role in the maintenance of glucose homeostasis.

## Open questions

1. Which RING-finger proteins are active regulators in PI3K/AKT-mediated glucose metabolism?
2. Which RING-finger proteins are negative regulators in PI3K/AKT-mediated glucose metabolism?
3. What is the exact mechanism of RING-finger ligase function in PI3K/AKT-mediated glucose metabolism?

## Introduction

Glucose is an important nutrient that provides most of the energy required by various organs to achieve homeostasis. Therefore, stability of glucose metabolism is crucial for maintaining organ function in humans [1]. However, the prevalence of high-glucose diets has increased attention on glucose metabolism-related disorders [2]. Abnormalities in glucose metabolism contribute to multiple health conditions, including obesity [3], diabetes mellitus [4], hypertension [5], cancer [6], and polycystic ovary syndrome [7]. Under aerobic conditions, tumor tissues in human cancers are more likely to utilize glucose to produce lactate instead of entering the mitochondria and tricarboxylic acid cycle, a phenomenon termed the Warburg effect [8]. Moreover, disorders related to glucose metabolism are associated with several complications. Extreme hyperglycemia might cause ketoacidosis, which alters blood vessels and the nervous system [9]. Phosphatidylinositol 3-kinase (PI3K)/AKT pathway-mediated glucose metabolism signaling, which promotes glycolysis and resists gluconeogenesis, plays a key role in cell biology. The PI3K/AKT pathway alters glucose metabolism, both directly and indirectly [10].

**Fig. 1:**
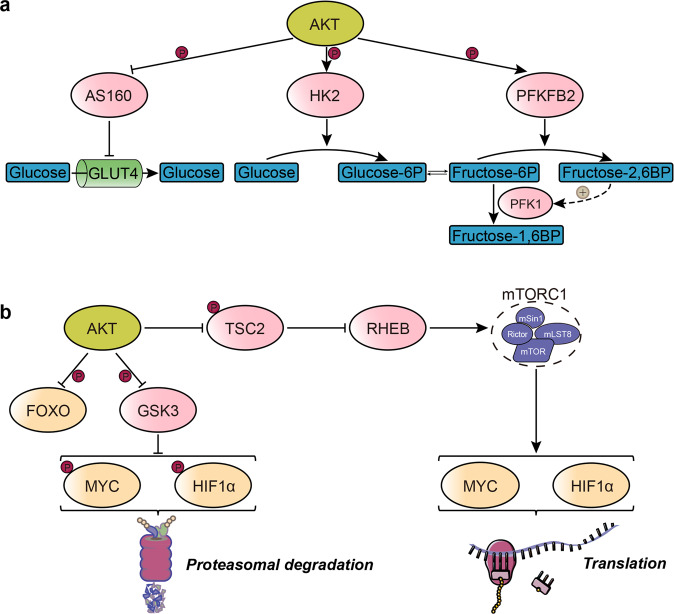
The direct or indirect regulations of AKT-mediated glucose metabolism. **a** AKT directly stimulates glucose metabolic changes by phosphorylating key glycolysis enzymes and influencing glucose transporters 4 translocation. **b** AKT indirectly changes glucose metabolism by impacting key downstream effectors through a combination of transcriptional, translational, and post-translational mechanisms. HK2: hexokinase 2; PFK1: phosphofructokinase-1; PFKFB2: fructose-2,6-biphosphatase; TSC2: tuberous sclerosis complex 2; RHEB: Ras homolog enriched in brain; mTORC1: mechanistic target of rapamycin complex 1; FOXO: forkhead box O; GSK3: glycogen synthase kinase 3; HIF1α: hypoxia-inducible factor 1α.

PI3K/AKT signaling, as a well-known pathway, is activated among many diseases, especially cancer [11] and diabetes mellitus [12]. It directly alters glucose metabolism via phosphorylation-associated regulation of metabolic enzymes as well as glucose transporters 4 (GLUT4) trafficking [13]. Such regulatory events rely substantially on PI3K/AKT-associated phosphorylation and suppression of AS160 [14]. In addition to glucose uptake, PI3K/AKT regulates critical glycolytic events by phosphorylating and activating various enzymes. Following entry into the cells, glucose is activated for metabolic use by hexokinases, which carry out glucose phosphorylation to generate glucose-6-phosphate, whose transport out of the cell by GLUT1 is impossible [15]. PI3K/AKT signaling also influences glycolysis by indirectly stimulating the activity of phosphofructokinase-1. Fructose-2,6- biphosphate is phosphorylated and activated by PI3K/AKT. This enzyme catalyzes the formation of fructose-2,6-biphosphate and activates phosphofructokinase-1, a rate-limiting glycolytic enzyme, thereby committing glucose carbons to glycolysis [16] (Fig. 1a).

PI3K/AKT directly phosphorylates multiple enzymes or modulators involved in metabolism or nutrient transport. It further induces major downstream effectors with significant roles in cell metabolic reprogramming, such as the mechanistic target of rapamycin complex 1 (mTORC1) and glycogen synthase kinase 3 (GSK3) [17]. PI3K/AKT signaling also sustains aerobic glycolysis by upregulating glucose transporters and glycolytic enzymes through the regulation of downstream transcription factors such as forkhead box O (FOXO)-related proteins and MYC [10]. This pathway primarily induces mTORC1 by phosphorylating and inhibiting tuberous sclerosis complex 2, an important part of the TSC complex. It does so by alleviating TSC complex-related suppression of a Ras homolog enriched in the brain [18]. GSK3, which blocks glycogen production by phosphorylating and suppressing its eponym substrate, is suppressed by growth factors and insulin through PI3K/AKT-related phosphorylation [19]. GSK3-associated phosphorylation of the transcription factors MYC and hypoxia-inducible factor 1α (HIF1α) promotes their proteasomal degradation [20, 21] (Fig. 1b). Previous studies have demonstrated that protein post-translational modifications play an essential role in PI3K/AKT-mediated glucose metabolism. Here, we summarize the underlying molecular mechanisms of RING E3 ligases in the regulation of PI3K/AKT-mediated glucose metabolism.

Protein ubiquitination is an important type of post-translational modification for ubiquitin-related protein degradation via the ubiquitin-proteasome system. The system comprises three enzymes: ubiquitin-activating enzyme (E1), ubiquitin-conjugating enzyme (E2), and ubiquitin ligase (E3). Under the condition of providing energy by ATP, ubiquitin-activating enzyme E1 catalyzes the activation of ubiquitin. The active ubiquitin is then transferred to ubiquitin-conjugating enzyme E2 and ubiquitin ligase E3, which covalently binds ubiquitin to a target protein [22]. Depending on the presence of characteristic domains, E3 ligases comprise three major families: RING (Really Interesting New Gene) family [23], HECT (homologous to the EA6P carboxyl terminus) domain family [24], and the U-box families [25]. RING-finger proteins are broadly found in eukaryotic cells [26] and are the most abundant of E3 ubiquitin ligases [27]. They perform functions by zinc-binding RING-finger adaptor or a U-box domain, which are responsible for binding the ubiquitin-conjugating enzyme E2 and promoting ubiquitin transfer. RING-finger E3 proteins control cellular events such as DNA damage response [28], cell signaling, and glucose homeostasis [29]. Therefore, abnormal expression of these proteins may result in multiple metabolic disorders. Cullin, tumor necrosis factor receptor-associated factor (TRAF), tripartite motif (TRIM), and Membrane-associated RING-CH-type finger (MARCH) proteins regulate PI3K/AKT-mediated glucose homeostasis. As such, comprehensive analysis of these versatile RING-finger E3s might help to develop new management tools for metabolic dysfunction. Previous studies have reported the role of ubiquitination in PI3K/AKT-mediated glucose metabolism. However, a review that highlights the relationship between RING-finger E3 ubiquitin ligase and PI3K/AKT-mediated glucose metabolism is still lacking. Here, we summarize the roles of RING-finger E3 ubiquitinated ligases, such as Cullin, TRAF and TRIM families in PI3K/AKT-mediated glucose metabolism.

## The Cullin Family and PI3K/AKT-mediated glucose metabolism

**Fig. 2:**
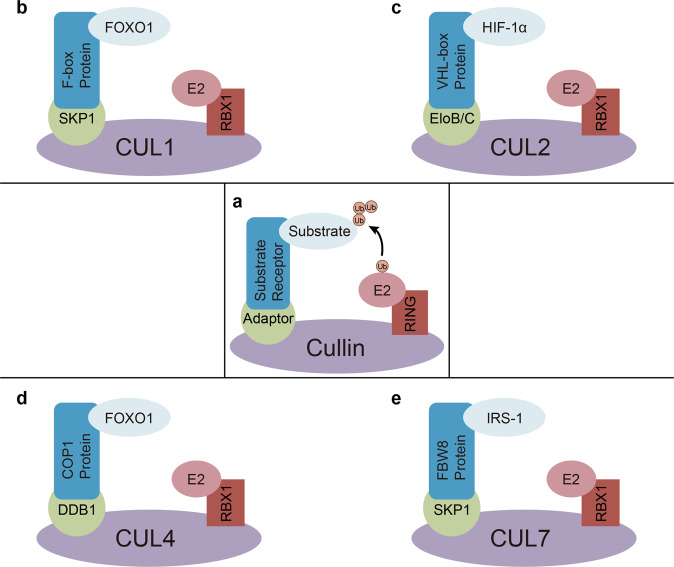
Architecture of the cullin-RING E3 ligase complex. **a** The components of a CRL. **b**–**e** Specific protein factors for each CRL family. *Skp1* S phase kinase-associated protein-1, *FOXO1* forkhead box O 1, *E2* ubiquitin conjugating enzyme, *Rbx1* regulator of cullins 1, *VHL* von Hippel-Lindau, *HIF1α* hypoxia-inducible factor 1α, *DDB1* damage-specific DNA binding protein 1, *COP1* constitutive photomorphogenesis protein 1, *Fbw8* F-box and WD-repeat-domain-containing protein 8, *IRS1* insulin receptor substrate 1.

Cullins, as molecular scaffolds, ubiquitinate via simultaneous interaction with a substrate and anchorage of an E2 ubiquitin-conjugating enzyme via the RING domain. They are also known as cullin-RING ligase complexes (CRLs) and constitutes the most abundant RING E3 ligases [30]. CRLs act via cognate substrate-recognition molecules, including F-box [31], suppressors of cytokine signaling (SOCS) [32], Bric-a-brac, Tramtrack, Broad-complex [33], and von Hippel-Lindau (VHL) proteins. These protein families comprise characteristic motifs recognized by specific adaptors conjugated to the respective cognate cullins. Therefore, a CRL brings a substrate near the E2 that transfers ubiquitin to the substrate [34] (Fig. 2a). Mammals have eight Cullin members, including CUL1, CUL2, CUL3, CUL4a, CUL4b, CUL5, CUL7 and PARC. CRL1, CRL2, CRL4, and CRL7 play important roles in PI3K/AKT-mediated glucose metabolism [29, 35–37].

### The CRL1 complex

**Fig. 3:**
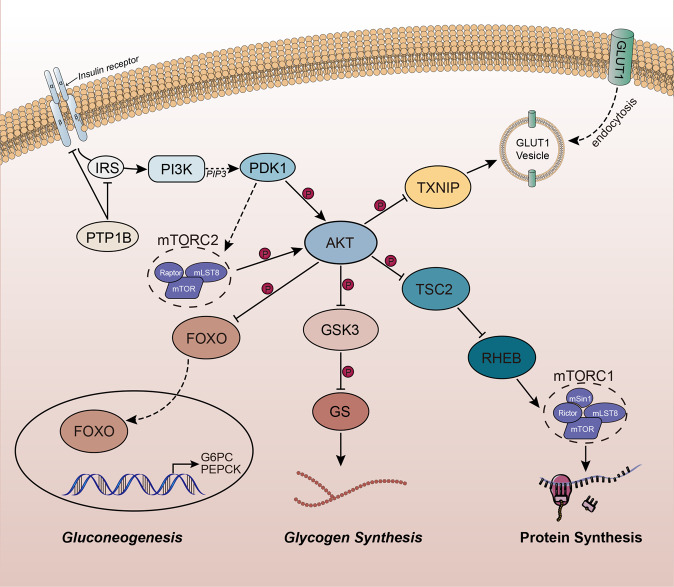
The PI3K/AKT signaling network. The upstream and major downstream effectors of AKT signaling in glycogen synthesis, gluconeogenesis, glucose transport, and protein synthesis. *IRS* insulin receptor substrate, *PI3K* phosphatidylinositol 3-kinase, *PTP1B* protein-tyrosine phosphatase 1B, *PIP3* phosphatidylinositol 3,4,5-trisphosphate, *PDK1* phosphoinositide-dependent protein kinase 1, *mTORC* mechanistic target of rapamycin complex, *FOXO* forkhead box O, *GSK3* glycogen synthase kinase 3, *TSC2* tuberous sclerosis complex 2, *RHEB* Ras homolog enriched in brain.

The multi-subunit CRL1 is a member of the RING E3 ligase family. It has broad substrate specificity and regulates multiple cellular events [38]. Most F-box proteins are involved in the formation of the SCF E3 ubiquitin ligase complex via Skp1, which interacts with CUL1 (Fig. 2b). Additionally, reports assessing F-box family members, such as β-TrCP, Skp2, F-box, and WD-repeat-domain-containing protein 7 (Fbw7), have demonstrated that a given substrate recognition protein can interact with many substrates, thus increasing the functional scope of CRLs and mediating ubiquitination and degradation of various signaling proteins [34]. The Skp2-SCF complex is an essential E3 ligase for PI3K/AKT ubiquitination and membrane recruitment. Skp2 suppression impairs PI3K/AKT activation, GLUT1 expression, glucose uptake, and glycolysis [35]. Moreover, the substrate-binding F-box protein Skp2 of the SCF-Skp2 E3 ligase binds equally to ubiquitinates and promotes the degradation of FOXO1 through PI3K/AKT-phosphorylated FOXO1 at Ser256 [39]. FOXO family members, including FOXO1, FOXO3A, and FOXO4, are canonical substrates of PI3K/AKT. They are phosphorylated and sequestered from the nuclear compartment, subsequently downregulating their respective target genes. FOXO1, the major hepatic FOXO protein that drives PEPCK and G6Pase expression, determines the ability of insulin to maintain glucose homeostasis and regulate glucose biosynthesis in the liver [40] (Fig. 3). By antagonizing the function of MYC, FOXO transcription factors inhibit the expression of glycolytic genes. The activated Skp2-SCF complex degrades FOXO1, thereby relieving the inhibitory effects of FOXO transcription factors on glycolytic enzymes and inhibiting gluconeogenesis [41] (Table 1).

**Table 1:** The functions and regulation of the Cullin family in PI3K/AKT-mediated glucose metabolism.

E3 ligases	Mechanisms	Biological response	References
CRL1	Skp2 deficiency impairs PI3K/AKT activation, GLUT1 expression, glucose uptake, and glycolysis. Skp2 can also ubiquitinate and promote the degradation of FOXO1. Downregulation of the PI3K/AKT-GSK3β-FBW7 signaling axis promotes the destabilization of c-Myc, which inhibit expression of hexokinase 2.	PI3K/AKT↑ Glycolysis↑ Gluconeogenesis↓	[35] [39–41] [42–50]
CRL2	CRL2 pVHL plays a critical role in the control of HIF1α degradation. HIF1α induces the expression of GLUT1 and nearly all glycolysis enzymes. The Cul2-Rbx1-SOCS complex ubiquitinates IRS1 and suppresses the activation of PI3K/AKT, which causes glucose metabolism changes.	Glycolysis↓ PI3K/AKT↓ Glycolysis↓	[21, 51–60] [61, 62]
CRL4	COP1, part of the Cul4A-RING E3 ubiquitin ligase complex, plays an important role in the homeostasis of glucose. It reduces PTP1B activity and suppresses the expression of gluconeogenic genes.	PI3K/AKT↑ Glycolysis↑ Gluconeogenesis↓	[37], [66–71]
CRL7	The SCF-Fbxw8 E3 ligase targets IRS-1 for degradation.	PI3K/AKT↓ Glycolysis↓	[29], [72–77]

*CRLs* cullin-RING ligase complexes, *Skp2* S phase kinase-associated protein-2, *PI3K* phosphatidylinositol 3-kinase, *GLUT1* glucose transporter 1, *FOXO1* forkhead box O 1; *FBW7* F-box and WD-repeat-domain-containing protein 7, *HIF1α* hypoxia-inducible factor 1α, *Rbx1* RING box protein 1, *SOCS* suppressors of cytokine signaling, *IRS1* insulin receptor substrate 1, *COP1* constitutive photomorphogenesis protein 1, *PTP1B* protein-tyrosine phosphatase 1B.

Fbw7, an F-box protein, is another substrate recognition protein of SCF ubiquitin ligase [42]. It promotes proteasome-dependent c-Myc ubiquitination in vivo and in vitro [43]. The MYC oncoprotein is an important transcription factor that regulates many genes involved in cell growth, proliferation, and metabolic pathways [44]. MYC, a transcription factor, induces the expression of GLUT1 and pyruvate kinase and hexokinase. In addition, MYC activity is generally decided by its abundance and is regulated via the way of transcriptional, translational mechanisms and PTM [45]. MTORC1 signaling regulates the abundance of MYC by enhancing the translation of MYC [46, 47], whereas PI3K/AKT promotes MYC stabilization by inhibiting proteasomal degradation. GSK3, a serine-threonine kinase, can phosphorylate MYC and facilitate its degradation [43, 48, 49] (Fig. 1b). Recent studies have suggested that downregulation of the PI3K/AKT-GSK3β-FBW7 signaling axis promotes destabilization of c-Myc, which downregulates hexokinase 2 [50] (Table 1).

### The CRL2 complex

The Elongin B/C-Cul2/Cul5-SOCS-box protein complex belongs to the RING E3 ubiquitin ligase family, which shares a Cullin-Rbx (RING box protein) module. SOCS-box proteins perform substrate recruitment to the complex and are associated with Cullin-Rbx through Elongin B/C (Fig. 2c). VHL is considered a SOCS-box protein, although it lacks the C-terminus of the SOCS box. It binds to endogenous Cul2-Rbx1 in mammalians [51]. CRL2pVHL is essential for controlling oxygen homeostasis via the regulation of hypoxia-inducing transcription factor for degradation [52]. HIF1α, whose degradation depends on oxygen levels, is stabilized under hypoxia. Furthermore, HIF1α upregulates GLUT1 and almost all glycolytic enzymes [53]. To adapt to hypoxic conditions, this protein also upregulates lactate dehydrogenase 1 and pyruvate dehydrogenase kinase 1, which promotes anaerobic glycolysis and inhibits oxidative phosphorylation [54]. Several reports have shown that PI3K/AKT signaling activates mTORC1, and further upregulates the expression of HIF1α, which upregulates gene targets such as phosphofructokinase, and enhances glucose uptake and glucose transformation into lactate, even in normoxia [55–58]. Thus, degradation of VHL enhances the function of HIF1α, which reprograms glucose and energy metabolism, including enhanced glucose uptake, glycolytic processes, and lactate synthesis [59, 60] (Table 1).

Many proinflammatory cytokines upregulate SOCS protein binding through the Src homology 2 domains to activate cytokine receptors or related Janus kinases, contributing to a negative feedback loop that attenuates cytokine signaling. The SOCS protein also interacts with the Elongin BC-containing E3 ubiquitin ligase complex via the SOCS box. SOCS1 and SOCS3 have been suggested to inhibit heterogeneous pathways by ubiquitinating and degrading insulin receptor substrate 1 (IRS1) and IRS2 [61], thereby suppressing insulin-associated phosphorylation of the p85 subunit of PI3K and AKT [62] (Table 1).

Phosphorylation of IRS by the insulation receptor at a tyrosine residue enables its interaction with Src homology 2 domain proteins, such as the regulatory subunits of class 1 A PI3K. PI3K activation at the cytoplasmic membrane induces phosphorylation of its phospholipid substrate, phosphatidylinositol 4,5-bisphosphate, to generate the second messenger, phosphatidylinositol 3,4,5-trisphosphate (PIP3) [63]. Threonine kinase AKT interacts with PIP3 and undergoes phosphorylation by phosphoinositide-dependent protein kinase 1 (PDPK1 or PDK1) at Thr308 or by mTORC2 at Ser473, which further induces the activation of AKT [64, 65] (Fig. 3). Further, PI3K/AKT signaling partly promotes cell proliferation by altering metabolic pathways in cells. In addition, ubiquitination of IRS1 mediated by the Cul2-Rbx1-SOCS complex suppresses the activation of PI3K/AKT, which causes changes in glucose metabolism.

### The CRL4 complex

Constitutive photomorphogenesis protein 1 (COP1), part of the Cul4A-RING E3 ubiquitin ligase complex, is an evolutionarily conserved protein [66]. COP1 can facilitate substrate degradation through its function of adaptor protein of other factors, including damage-specific DNA binding protein 1 (DDB1), CUL4 and Rbx1, to form a large complex [67] (Fig. 2d). Plant studies have deeply assessed COP1, which is also expressed in mammalian organisms, controlling gluconeogenesis, lipid metabolism and tumorigenesis [68]. This protein affects glucose homeostasis by downregulating gluconeogenic genes, thereby suppressing glucose production in the liver [37]. In the presence of insulin, COP1 can induce IRS phosphorylation by inhibiting the activity of protein-tyrosine phosphatase 1B (PTP1B), which is a negative regulator of insulin receptor and IRS [69] (Fig. 3). The regulator mechanism activates multiple protein kinases that reduce hepatic glucose biosynthesis, thereby maintaining glucose homeostasis [70]. Moreover, insulin upregulates COP1, which interacts with FOXO1 and induces its degradation through the ubiquitin-proteasome system [37, 71]. In this manner, the CUL4A-DDB1-COP1 complex inhibits gluconeogenesis and maintains glucose homeostasis (Table 1).

### The CRL7 complex

CUL7 belongs to the cullin family of RING E3 ubiquitin ligases and interacts with the Rbx1 RING-finger protein to generate a complex. In this complex, Fbxw8 and Fbxw11 are the only F-box proteins that interact with CUL7 [72]. Several studies have shown that SCF-Fbxw8 E3 ligase targets IRS-1 for degradation. However, the mechanism employed by CUL7 to selectively interact with Fbw8 is unclear. CUL7 has been proven to resemble CUL1 in utilizing the Skp1 adaptor [73] (Fig. 2e). In addition, CUL7 suppression in the C2C12 cell line and heterozygous knockout of mouse CUL7 or Fbxw8 elevated IRS-1 protein levels, PI3K/AKT activity, and glucose uptake after insulin stimulation [29, 74]. These findings confirm that CUL7 modulates the insulin pathway and glucose homeostasis. The function of Fbw8 is regulated by mTORC2, upstream of AKT, which leads to the phosphorylation and activation of AKT [75]. MTORC2 induces Fbw8 stabilization via phosphorylation at Ser86, promoting insulin-associated cytoplasmic translocation of Fbw8 with subsequent IRS1 degradation [76]. The CD36 receptor functions via ubiquitination-dependent interaction with IRS1, suppressing binding to CUL7. Furthermore, Fyn (a Src family kinase) dissociation from CD36 using free fatty acids or Fyn silencing/suppression accelerates insulin-associated IRS1 degradation. This is possibly due to the inhibition of IRS1 binding to CD36, which enhances the interaction with CUL7. This process is critical for the negative feedback modulation of insulin pathway induction and glucose metabolism [77] (Table 1).

## The TRAF Family and PI3K/AKT-mediated glucose metabolism

TRAF domain, which is subdivided into two regions: the TRAF-N domain and TRAF-C domain, is a characteristic feature of TRAF family. At the N-terminal, most TRAFs possess a RING domain, followed by many zinc finger domains [78], and various intracellular signaling molecules bind to the TRAF-N domain [79]. The TRAF family serves as adaptor proteins that mediate cytokine signaling and overtly regulate cell survival, proliferation, and stress response [80]. TRAF6 and TRAF4, which are members of the TRAF family, regulate multiple protein-to-protein interactions through the TRAF and RING-finger domains with nonconventional E3 ubiquitin ligase activity [81]. Ubiquitin binds via a lysine residue (K63, K48, K33, K29, K27, K11, or K6) or N-terminal methionine, allowing the assembly of specific polymers [82], although K48 and K63 are the greatest players. K48, K11, and K29-linked poly-Ub directly interact with substrates for proteasomal degradation [83, 84], whereas mono-ubiquitination and K63-associated poly-Ub chains play nonproteolytic roles [85]. Interestingly, TRAF4 and TRAF6-mediated ubiquitination of AKT act via K63-induced ubiquitination, but not K48-dependent ubiquitination, which has no effect on AKT stability but controls PI3K/AKT pathway activation by promoting the AKT membrane recruitment and phosphorylation by serine/threonine kinase [86–88].

TRAF6-mediated membrane recruitment and AKT induction may occur via K63-linked ubiquitination of APPL1. APPL1, an adaptor protein, binds to AKT and suppresses AKT binding to its endogenous suppressor tribble 3 via direct competition, thereby inducing AKT translocation to the cytoplasmic membrane and endosomes for subsequent activation [89–91]. K63-linked ubiquitination of APPL1, which is induced by TRAF6, enhances membrane recruitment and activation of Akt. A recent study showed that TRAF6 downregulation alleviates insulin-induced ubiquitination and membrane targeting of APPL1, impairing insulin-induced PI3K/AKT activation [92]. In addition, histone lysine demethylase 4B promotes glucose uptake by regulating GLUT1, which is ubiquitously expressed and controls cellular glucose uptake. Histone lysine demethylase 4B interacts with TRAF6 and promotes PI3K/AKT ubiquitination and activation [93]. Furthermore, thioredoxin-interacting protein (TXNIP), a downstream substrate of PI3K/AKT, contributes to the control of GLUT1 trafficking [94]. The protein enhances GLUT1 endocytosis and suppresses glucose uptake [95]. PI3K/AKT signaling phosphorylates and suppresses TXNIP, rapidly increasing GLUT1 and GLUT4 levels in the cytoplasmic membrane and enhancing cellular glucose uptake [96] (Fig. 3). Furthermore, studies have indicated that TRAF4 was overexpressed in human lung cancer cells and tissues and is necessary for activating the critical cell survival kinase PI3K/AKT via ubiquitination [97]. These studies demonstrated that PI3K/AKT undergoes K63-associated ubiquitination by TRAF6 and TRAF4, which is critical for PI3K/AKT membrane recruitment and facilitates glycolysis and glucose uptake [86, 88, 97] (Table 2).

**Table 2:** The functions and regulation of TRAF, TRIM, and MARCH families in PI3K/AKT-mediated glucose metabolism.

E3 ligases	Mechanisms	Biological response	References
TRAF6	The suppression of TRAF6 expression attenuates insulin-mediated ubiquitination and membrane targeting of APPL1, leading to an impairment of insulin stimulated PI3K/AKT activation.	PI3K/AKT↑ Glycolysis↑	[86–93]
TRAF4	TRAF4 is required for activation of the pivotal cell survival kinase PI3K/AKT through K63-linked ubiquitination.	PI3K/AKT↑ Glycolysis↑	[86, 97]
TRIM31	Decreased AKT Thr308 phosphorylation was observed in TRIM31-KO mice, suggesting that TRIM31 promotes AKT phosphorylation. TRIM31 also actives mTORC1, which is conducive to glycolysis and glucose uptake.	PI3K/AKT↑ Glycolysis↑	[102, 104]
TRIM32	TRIM32 promotes the growth of gastric cancer cells by enhancing AKT activity and glucose transportation.	PI3K/AKT↑ Glycolysis↑	[103–106]
TRIM63	TRIM63, is a negative regulator of PI3K/AKT. MuRF1-KO mice have elevated AKT-Ser-473 activation.	PI3K/AKT↓ Glycolysis↓	[107, 109]
TRIM26	Overexpression of TRIM26 inhibited the activation of PI3K/AKT pathway and caused significant decrease in glucose uptake and lactate production in papillary thyroid carcinoma cells.	PI3K/AKT↓ Glycolysis↓	[108]
MARCH5	MARCH5 increases aerobic glycolysis and lactate production through the modulation of cellular growth factor-binding receptor tyrosine kinase endocytosis, which leads to increased PI3K/AKT activation.	PI3K/AKT↑ Glycolysis↑	[115]

*TRAF* tumor necrosis factor receptor-associated factor, *PI3K* phosphatidylinositol 3-kinase, *TRIM* tripartite motif, *MuRF1* muscle-specific RING finger protein-1.

## The TRIM family and PI3K/AKT-mediated glucose metabolism

TRIM-related proteins are E3 ubiquitin ligases containing a RING-finger domain. However, not all proteins with a RING-finger domain may act as E3 ubiquitin ligases [98]. In addition to the RING-finger domain, TRIM proteins have one or two zinc-binding motifs termed B-boxes and a coiled-coil region [99]. Multiple substrate specificities of TRIM proteins may be determined by shuffling the respective ligands [100]. Most TRIM proteins, which act as E3 ubiquitin ligases can contribute to many oncogenic events, including transcriptional regulation, cell proliferation, apoptosis, and tumorigenesis [101].

Several studies have demonstrated that TRIM proteins regulate glucose homeostasis by increasing PI3K/AKT induction and glucose transportation [102, 103]. TRIM31-knockout (KO) mice developed glucose intolerance and insulin resistance. Furthermore, decreased AKT Thr308 phosphorylation has been detected in TRIM31-KO mice [102]. TRIM31 also exerted its function by directly interacting with the tuberous sclerosis complex and promoting the degradation of this complex, the upstream suppressor of mTORC1, which facilitates the glycolysis and glucose uptake [104] (Table 2). TRIM32, another TRIM protein, represents an E3 ubiquitin ligase [105]. TRIM32 levels are elevated in many cancers, promoting malignant cell proliferation [106, 107]. Furthermore, TRIM32 is a proliferation and anti-apoptotic protein involved in PI3K/AKT signaling in gastric cancer. This protein potentially controls glycolysis by upregulating GLUT1 and hexokinase 2 in gastric cancer cells [103] (Table 2).

However, some TRIM proteins are considered as negative regulators that suppress the phosphorylation of PI3K/AKT [108, 109]. TRIM63, also known as muscle-specific RING-finger protein-1 (MuRF1), plays an important role in skeletal and cardiac muscle atrophy. This protein is a negative regulator of PI3K/AKT [110]. MuRF1-KO mice showed higher serum glucose and triglyceride levels, and decreased glucose tolerance. MuRF1-KO skeletal muscle had an altered PI3K/AKT pathway, enhanced AKT-Ser-473 induction, and decreased oxidative mitochondrial function, suggesting possible mechanisms underlying MuRF1-associated modulation of glucose and fat metabolism (Table 2). Meanwhile, MyoMed-205, the MuRF1 inhibitor in mice with experimental diabetes significantly affects serum glucose [108]. TRIM26 acts as a tumor suppressor in multiple forms of cancer [111]. A recent study investigated the effect of this protein on proliferative, metastatic, and glycolytic processes in papillary thyroid carcinoma (PTC). TRIM26 overexpression was found to inhibit the malignant potential of PTC cells and substantially reduce glucose uptake and lactate synthesis. Further studies demonstrated that TRIM26 overexpression inhibited PI3K/AKT signaling (Table 2). Administration of an inducer (740Y-P) of PI3K/AKT signaling reversed the anticancer effects of TRIM26 in PTC cells. These data provide evidence that TRIM26 acts as a negative regulator of PI3K/AKT in PTC cells [109]. TRIM proteins exhibit both the biological effects of upregulation and downregulation of PI3K/AKT signaling, thus playing different roles in cell metabolism.

## The MARCH Family and PI3K/AKT-mediated glucose metabolism

MARCH proteins, as a subfamily of the RING-finger E3 ligases, contains a C4HC3-type RING domain. K3 and K5, MARCH E3 ubiquitin ligases that are expressed by Kaposi’s sarcoma-associated herpesvirus, are the initial membrane-associated ubiquitin E3 ligases [112] and with further researches, more other members which have RING-CH domains were identified [113]. On the mitochondrial outer membrane, MARCH5 is ubiquitously expressed in human organs such as the heart, brain, liver, and lungs. MARCH5 has four transmembrane domains and an N-terminal C4HC3-type RING-finger domain, which is important for ubiquitin ligase activity [114]. In an earlier study, the authors found that besides a potential effect on antiviral immune responses [115], MARCH5 equally enhances proliferation and alters metabolism in cells [116]. This ubiquitin ligase enhances aerobic glycolysis and lactate biosynthesis by regulating cell growth factor-binding receptor tyrosine kinase endocytosis, thereby enhancing cell sensitivity to autocrine and paracrine factors, altering the pattern of cell phosphorylation, and increasing PI3K/AKT activation [117] (Table 2).

## Conclusion

By organizing RING-finger proteins that direct many substrates for ubiquitin-associated degradation or K63-mediated nonproteolytic functions, RING family proteins generate an important regulatory network that controls glucose homeostasis via the PI3K/AKT pathway, thereby regulating multiple biological processes. This review summarized the roles and regulation of RING-finger proteins in association with PI3K/AKT-mediated glucose metabolism. Among the zinc-binding RING-finger adaptor proteins, CRLs, TRAF, TRIM, and MARCH are involved in maintaining glucose homeostasis.

In recent years, the association of RING family members, especially CRLs and TRAF, with metabolic disorders have received extensive attention. The PI3K/AKT pathway is a classical signaling transduction pathway that is related to cell metabolism. Exploring the relationship between RING-finger proteins and PI3K/AKT-mediated glucose metabolism would provide new insights into the treatment of metabolic diseases.

## Data Availability

The data used to support the findings of this study are available from the corresponding author upon request.
